# Locally Resonant Phononic Crystals at Low frequencies Based on Porous SiC Multilayer

**DOI:** 10.1038/s41598-019-51329-z

**Published:** 2019-10-14

**Authors:** Ahmed Mehaney, Ashour M. Ahmed

**Affiliations:** Physics Department, Faculty of Science, Beni-Suef University, Beni-Suef, 62514 Egypt

**Keywords:** Engineering, Physics

## Abstract

In this work, a one-dimensional porous silicon carbide phononic crystal (1D-PSiC PnC) sandwiched between two rubber layers is introduced to obtain low frequency band gaps for the audible frequencies. The novelty of the proposed multilayer 1D-PnCs arises from the coupling between the soft rubber, unique mechanical properties of porous SiC materials and the local resonance phenomenon. The proposed structure could be considered as a 1D acoustic Metamaterial with a size smaller than the relevant 1D-PnC structures for the same frequencies. To the best of our knowledge, it is the first time to use PSiC materials in a 1D PnC structure for the problem of low frequency phononic band gaps. Also, the porosities and thicknesses of the PSiC layers were chosen to obtain the fundamental band gaps within the bandwidth of the acoustic transducers and sound suppression devices. The transmission spectrum of acoustic waves is calculated by using the transfer matrix method (TMM). The results revealed that surprising low band gaps appeared in the transmission spectra of the 1D-PSiC PnC at the audible range, which are lower than the expected ones by Bragg’s scattering theory. The frequency at the center of the first band gap was at the value 7957 Hz, which is 118 times smaller than the relevant frequency of other 1D structures with the same thickness. A comparison between the phononic band gaps of binary and ternary 1D-PSiC PnC structures sandwiched between two rubber layers at the micro-scale was performed and discussed. Also, the band gap frequency is controlled by varying the layers porosity, number and the thickness of each layer. The simulated results are promising in many applications such as low frequency band gaps, sound suppression devices, switches and filters.

## Introduction

Recently, the study of acoustic waves propagation in artificially structured materials has a great interest due to their novel applications in acoustic devices. The PnC structure is considered as a periodic arrangement of alternatively elastic/acoustic structures that exhibit stop bands or the so-called (phononic band gaps)^1^. Over these bands, all incident acoustic waves cannot propagate through the structure and effectively attenuated. In other words, the PnCs can play the role of elastic/acoustic mirrors in the frequency range of the phononic band gaps. Therefore, the PnCs are used to control the propagation of acoustic and elastic waves in a large scale of elastic/acoustic waves frequencies^2^. Band gaps caused by a periodic structure have been observed in semiconductors for electron waves and in photonic crystals for light waves^3–6^. The PnC provides a wide range of applications such as acoustic filter, sound isolation, acoustic waveguides, acoustic laser, acoustic diode, high-resolution acoustic imaging devices, noise suppression devices, RF communication, acoustic resonators, sensors, acoustic mirrors, switches, lenses, and transducers^7–14^.

Many PnCs were designed to control the motion of elastic/acoustic waves (phonons) with frequencies ranging from sound to heat^15^. The sound wave is basically made up of acoustic waves with frequencies roughly between 20 Hz and 20 kHz, or wavelengths ranging from meters to several tens of centimeters. Most of the previous investigations on the conventional PnCs depend on the Bragg’s scattering theory for noise control engineering^16,17^. The behavior of acoustic waves propagation thought PnCs depends on the ratio between their scattering wavelength and the lattice parameter of PnCs^18^. The frequency at the center of the band gap is inversely proportional to the lattice parameter $1/{\rm{\omega }}\propto {\rm{\lambda }}\propto {\rm{a}}$. Unfortunately, periodic structures need to create a phononic band gap in the sonic regime and act as sonic mirrors must have lattice constants with several meters. Hence, the PnC can hardly satisfy the requirement of low-frequency band-gap of PnCs with small dimensions. Liu *et al*. introduced a pioneering work for the locally resonant mechanism in 3D PnCs^19^. Similar works by Goffaux *et al*. and Wang *et al*. showed that narrow band-gaps at low frequencies can exist in its 2D and 1D counterparts^20,21^.

On the one hand, the porous PnCs (PPnCs) with air void embedded in the solid matrix have exceptional advantages over other ordinary PnCs. These materials are low mass density, high mechanical properties and good absorber for sound energy. The porous materials contain a large number of interconnected pores which determine the acoustical performance of the material. The tunability of porosity means that the physical properties of the porous layers are also tunable. Hence, the speed of sound, density and Young’s modulus of the porous materials can be controlled by changing the porosity ratio and the filling medium. Therefore, these materials can make strong sound localization greater than the other ordinary PnC soft materials. Moreover, the porous PnCs can be very light-weighted and easily fabricated with low manufacturing cost. Most of the previous work focused on the wave propagation in porous silicon (PSi) PnCs^22,23^. Their results show that unique behavior over for the bulk material depending on the porosity. Gazi and Bernhard fabricated a 1D Fibonacci PnC from PSi by electrochemical etching^23^. The transmittance of the longitudinal acoustic wave was studied in the 0.1–2.6 GHz range. Acoustic band gaps deeper than 50 dB were detected in the structure. Lazcano *et al*. studied experimentally and theoretically the localization of acoustic modes in periodic PSi structure in the GHz range^24^. Many localized modes appeared in the band gap coming from the defect layers inserted into the periodic structure. Saeid *et al*. manufactured porous PnC plate (PPCP) by laser cutting a Plexiglas (PMMA) plate^25^. Low frequency wide complete band gaps of plate waves were observed in the structure spectrum.

On the other hand, the porous silicon carbide (PSiC) is a particular type of ceramic matrix composite materials that are made up of ceramic fibers or particles lie in a ceramic matrix phase. It exhibits excellent properties of both porous ceramics and silicon carbide. It has amazing interest properties including low thermal expansion, high-temperature melting, high thermal conductivity and high hardness, low density, high specific strength, high permeability, excellent catalytic activity, high oxidation resistance and superior chemical inertness^26,27^. The PSiC has significant advantages over than PSi due to its exceptional mechanical and chemical properties. It possesses greater chemical resistance allows it to be used for biological applications without any additional coating required^28^.

In the present work, the phononic band gaps of acoustic waves in the low frequency range are studied based using multilayers from PSiC sandwiched between two rubber layers at the micro-scale. A comparison between conventional PnC and PSiC multilayer is addressed. Also, the effects of the binary and ternary 1D-PSiC PnC on the band gap width and on the transmission modes are introduced and discussed. The transmission spectrum of the acoustic waves is calculated using the TMM. The frequency band structure is controlled by varying the porosity, number of layers and the thickness of each of the layer.

## PnC Design

**Figure 1:**
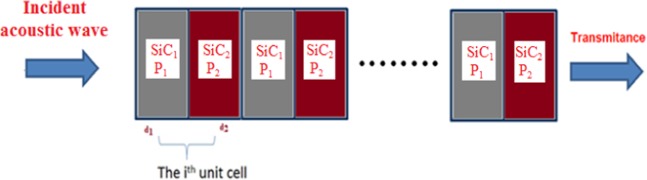
A schematic diagram of a 1D binary porous PnC consists of (SiC_1_/SiC_2_)^n^.

To have an idea about the effect of the acoustic wave at low frequencies on the transmission spectra of 1D-PSiC PnCs, a binary and ternary PnC from a multilayer of PSiC is addressed. The modeled structure was based on the following configurations: rubber/(PSiC_1_/PSiC_2_)^n^/rubber and rubber/(PSiC_1_/PSiC_2_/PSiC_3_)^n^/rubber. The effects of the period number n and the thickness of rubber layers were investigated. A schematic diagram of the (PSiC_1_/PSiC_2_)^n^ structure is shown in Fig. 1. For comparison, a 1D-PnC composed of alternately conventional epoxy and lead layers were studied. The porosities and thicknesses of the PSiC layers were chosen to obtain the fundamental stop band within the bandwidth of the acoustic transducers and sound suppression devices. The physical thicknesses, density and longitudinal sound velocity of each layer that used in this study are given in Table 1.

**Table 1:** The values of the acoustic parameters of each layer used in the study^49,71–73^.

Material Porosity ratio P (%)	Density (kg/m^3^)	Longitudinal sound speed (m/s)	Thickness (mm)
PSiC_1_ (14.41)	2550	9870	4
PSiC_2_ (31.65)	2030	7990	1
PSiC_3_ (20.87)	2350	9360	4
Epoxy (0)	1180	2535	4
Lead (0)	11400	1960	1
Rubber (0)	1300	22.87	From 0 to 4

## Theoretical Model

Several methods have been developed to investigate the elastic/acoustic waves propagation in PnCs include the transfer matrix method (TMM), the plane wave expansion (PWE) method, the finite difference time domain (FDTD) method, and the multiple-scattering theory (MST)^29–33^. The TMM has been widely used to calculate exact elasticity solutions for the transmission-frequency spectrum in 1D structures.

For an acoustic wave propagating normally through a 1D multilayer structure from the left to the right as shown in Fig. 1, the wave equation is given by the following relation^16,34^

1 $${\nabla }^{2}{\rm{\phi }}-{{\rm{c}}}_{{\rm{j}}}^{-2}\,\ddot{{\rm{\phi }}}=0,$$

where φ is the displacement and c_j_ is the acoustic wave velocity in each layer. The subscript j = 1, 2 and 3 represents the layer type.

The solution of Eq. 1 can be written as a superposition of transmitted and reflected traveling waves with harmonic time dependence^35^

2 $${\rm{\phi }}=({{\rm{A}}}_{1}\,{{\rm{e}}}^{+{\rm{i}}{{\rm{k}}}_{{\rm{j}}}{\rm{x}}}+{{\rm{A}}}_{2}\,{{\rm{e}}}^{-{\rm{i}}{{\rm{k}}}_{{\rm{j}}}{\rm{x}}}){{\rm{e}}}^{-{\rm{i}}{\rm{w}}{\rm{t}}},$$

where ${\rm{i}}=\sqrt{-1}$ is a complex number, k_j_ = 2πf/c_j_ is the wavenumber and f is the frequency of the incident wave.

The first and second terms of Eq. (2) represent the transmitted and reflected waves, respectively^36^. The coefficients A_1_ and A_2_ are the amplitudes of transmitted and reflected waves, respectively^23,37^.

By using the analysis of the TMM and applying the continuity condition at interfaces between layers, we deduce the following two wave matrices^38–40^.

3 $${{\rm{K}}}_{{\rm{ij}}}=\frac{1}{2}[\begin{array}{cc}\frac{{{\rm{Z}}}_{{\rm{j}}}+{{\rm{Z}}}_{{\rm{i}}}}{{{\rm{Z}}}_{{\rm{j}}}} & \frac{{{\rm{Z}}}_{{\rm{j}}}-{{\rm{Z}}}_{{\rm{i}}}}{{{\rm{Z}}}_{{\rm{j}}}}\\ \frac{{{\rm{Z}}}_{{\rm{j}}}-{{\rm{Z}}}_{{\rm{i}}}}{{{\rm{Z}}}_{{\rm{j}}}} & \frac{{{\rm{Z}}}_{{\rm{j}}}+{{\rm{Z}}}_{{\rm{i}}}}{{{\rm{Z}}}_{{\rm{j}}}}\end{array}],$$

4 $${{\rm{K}}}_{{\rm{j}}}=[\begin{array}{cc}{{\rm{e}}}^{-{{\rm{ik}}}_{{\rm{i}}}{\rm{d}}} & 0\\ 0 & {{\rm{e}}}^{-{{\rm{ik}}}_{{\rm{i}}}{\rm{d}}}\end{array}].$$

where Z is the characteristic impedance of layers, Z = ρv, from this relation the impedance depends on the density ρ of the layer and the acoustic velocity v, which in turn depends on the porosity of each layer. Equations (3) and (4) represent the wave matrix at the interface between the two layers and the wave matrix through each layer, respectively.

The relationship between the incident φ_0_ and transmitted φ_N_ wave state vectors are written as follows: ${{\rm{\phi }}}_{0}={K{\rm{\phi }}}_{{\rm{N}}}$ and K is the accumulative transfer matrix. We can calculate the transmission coefficient of the 1D PnC structure using the following relation:

5 $${\rm{T}}=1/{|{{\rm{K}}}_{11}|}^{2}.$$

where K_11_ is the first element of the accumulative matrix K.

## Results and Discussion

### Comparison between lead/epoxy and PSiC_1_/PSiC_2_ PnCs

**Figure 2:**
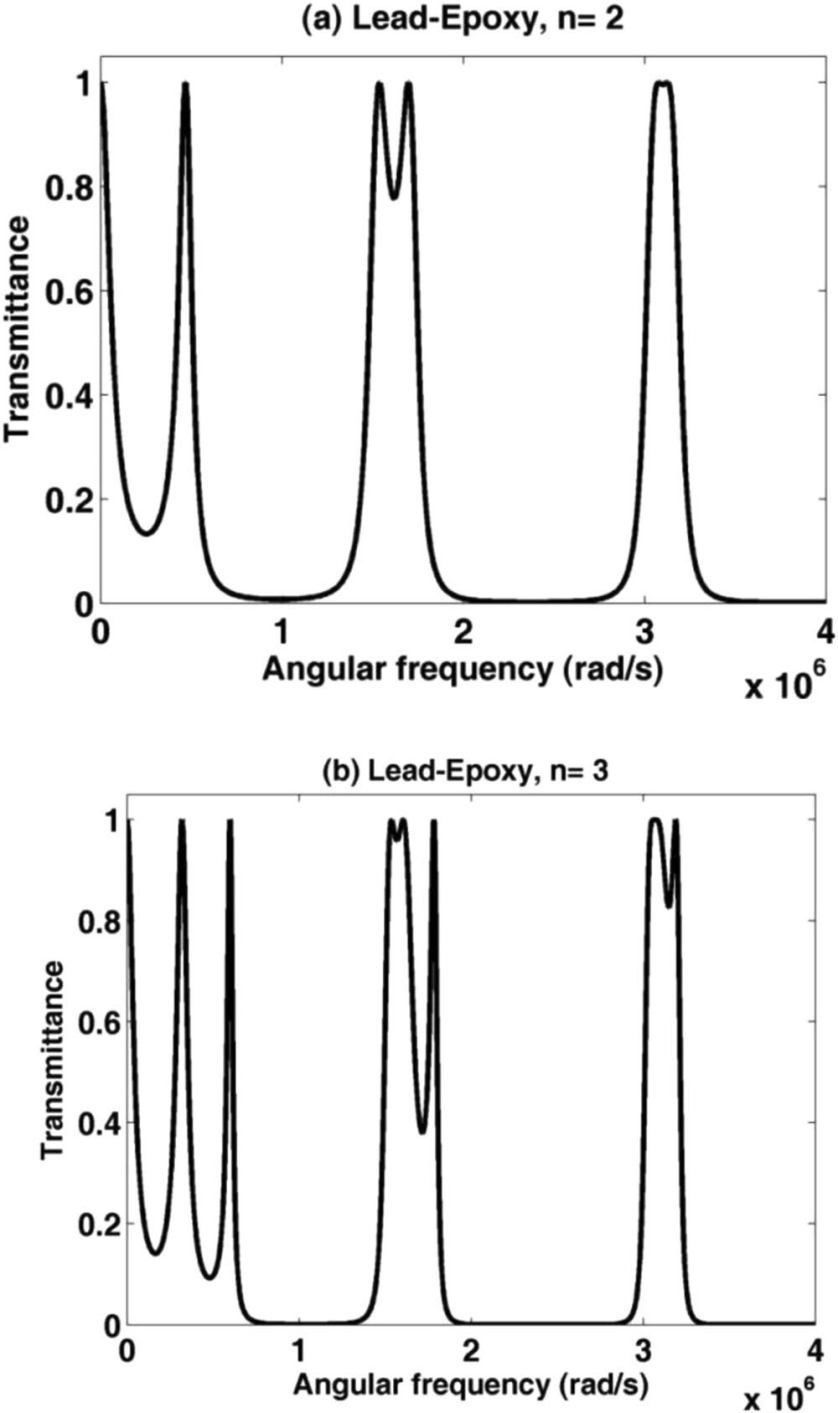
The transmission spectrum of a binary PnC composed of (lead/epoxy) with periods (**a**) n = 2 and (**b**) n = 3.

**Figure 3:**
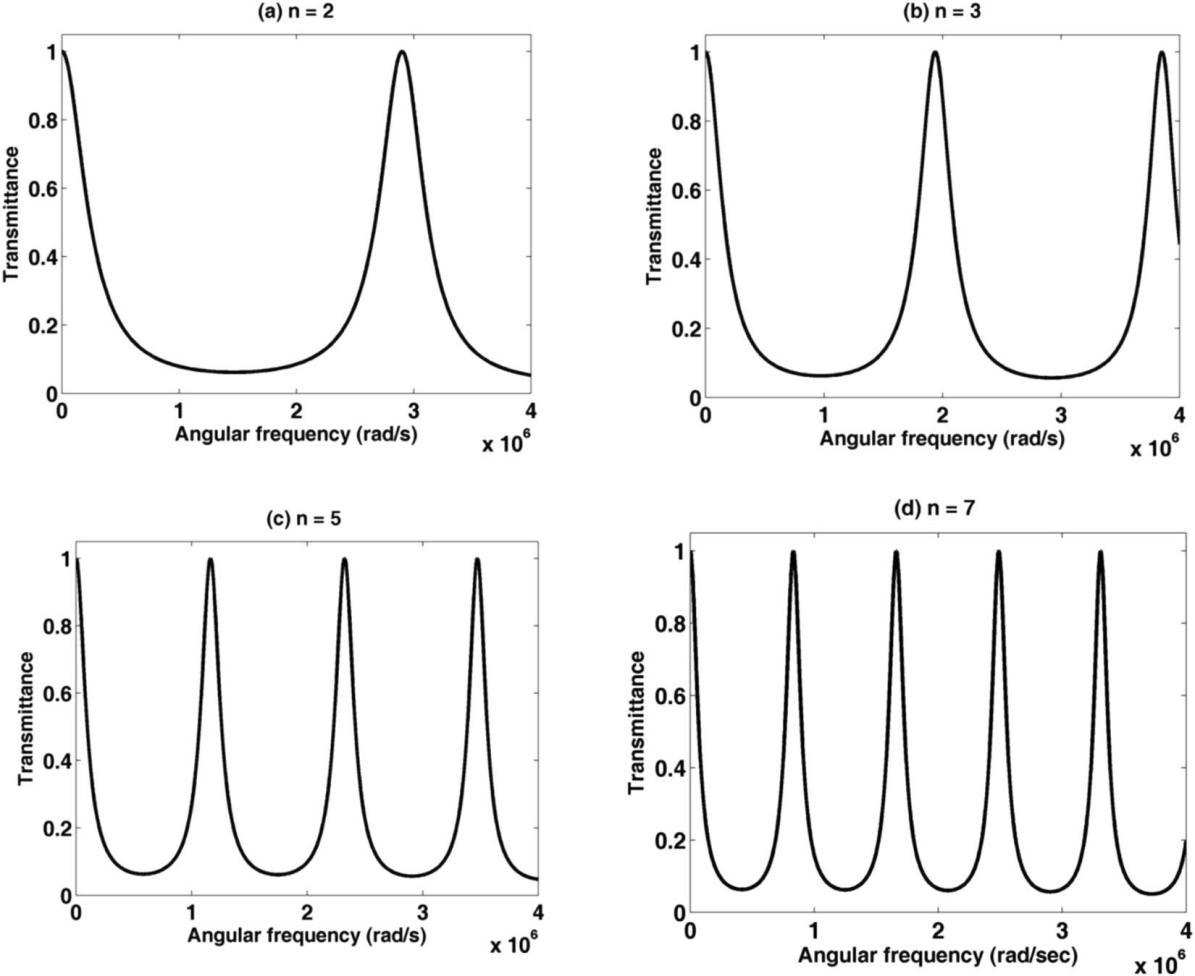
The transmission of a (PSiC_1_/PSiC_2_)^n^ PnC with porosity ratios 14.41% and 31.65%, respectively, at different periods (**a**) n = 2, (**b**) n = 3, (**c**) n = 5 and (**d**) n = 7.

As shown in Figs 2 and 3, the transmission spectra of two binary PnCs with an identical geometrical structure and different materials are plotted as a function of the angular frequency. The first PnC structure consists of lead/epoxy layers while the second PnC consists of PSiC_1_/PSiC_2_ layers with porosity ratios of 14.41% and 31.65%. In Fig. 2, the transmission spectrum is characterized by three different band gaps in the ultrasonic range, through them the acoustic waves cannot propagate through the entire structure. These regions (stop bands) resulted from the constructive interference between the incident and reflected acoustic waves at the interfaces of the constituent^37,41^. With increasing the number of periods from 2 to 3, a little change in the transmission spectrum occurred and no significant influence in the band-gap is observed. Also, there is no transmission modes (resonant modes or peaks) appeared in the entire spectrum or inside the band gaps of the lead/epoxy PnC structure. In the other PnC structure, the transmission spectra for binary (PSiC_1_/PSiC_2_)^n^ PnC are shown in Fig. 3. The porosity ratios of PSiC_1_/PSiC_2_ layers are 14.41% and 31.65%, respectively. The thickness of the layers PSiC_1_ and PSiC_2_ are 1 and 4 mm, respectively. The number of periods is varies from 2 to 7.

For n = 2, the band gap becomes wider and occupies the entire spectrum. Increasing the periodicity number inside the PnC structure leading to the creation of more interfaces between the periodic materials. Therefore, the band gaps appear clearer with increasing the periodicity number n. Also, the number of phononic band gaps increase with increasing the periodicity number n. The high mismatch in elastic properties between voids and solid material in the porous structure increases the width of the phononic band gaps as well.

The values of the transmission for all (PSiC_1_/PSiC_2_)^n^ PnC in many certain regions of frequencies are less than 10%. This value is decreased with increasing the periodicity number. Therefore, the transmission of the wave is very small through the PnC within these regions, which can be referred to it as phononic band gaps (PnBGs). In many previous articles, the transmission of the wave within the phononic band gaps can be considered from the 80% drop of its maximum value (100%)^42–45^. The high-velocity mismatch between elastic solids and voids (large enough difference in the acoustic impedances) is enough sufficient to produce wide phononic band-gaps^46,47^.

To sum up, we can say that the wave propagation behaviors of PSiC_1_/PSiC_2_ is totally different compared with lead/epoxy PnCs due to the highest change in the sound velocity and density of voids and the bulk solid material. Hence, it can control the values of elastic constants in order to fabricate different PnCs rather than the constant values of the other materials. This feature is the unique merit of porous materials than the conventional ones besides they are all produced from single bulk material. By using the porous materials, we can produce and study distinct properties (e.g., density, speed, ….) original materials do not have. With the same bulk PSiC material we can produce layers having very high and very low constant & density.

### Band structure of PSiC PnC

**Figure 4:**
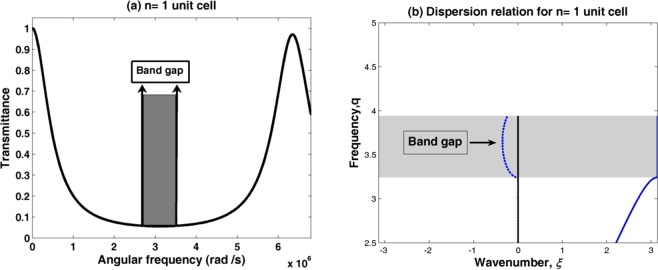
(**a**) The transmission spectrum and (**b**) dispersion curve of acoustic waves propagation through (PSiC_1_/PSiC_2_)^1^ with thicknesses 4 mm and 1 mm, respectively.

The band structure of PSiC PnC is calculated for wave propagation through an infinite structure based on Bloch-Floquet theorem^38,48^. Figure 4 shows the transmission spectrum and the band structure of acoustic wave propagation through the SiC PnC structure by using one unit cell (n = 1) from the binary (SiC_1_/SiC_2_) structure with thickness 4 mm and 1 mm, respectively.

The dispersion relation (band diagram) describes the wave propagation characteristics through a periodic medium. The dispersion curve is plotted between the non- dimensional wave frequency q (q range is $2.5\le {\rm{q}}\le 5$ with ${\rm{q}}={{\rm{q}}}_{{\rm{LB}}}={\rm{\omega }}{\rm{a}}/{{\rm{c}}}_{{\rm{LB}}}$) for longitudinal waves versus the non- dimensional wavenumber ${\rm{\zeta }}={\rm{k}}\times {\rm{a}}$. Where k is the incident wavenumber, a is the unit cell length, c_LB_ is the longitudinal wave velocities in the second material (SiC_2_). The frequency range was discretized into 1001 sample points. The details analysis of the dispersion relation of elastic/acoustic wave propagation through 1D PnC structures can be found in many literatures^49,50^.

The obtained dispersion relation is a completely Eigen problem that relates Eigenvalues with Eigenvectors as follows

6 $${{\rm{V}}}_{2{\rm{R}}}^{(i)}={{\rm{V}}}_{2{\rm{R}}}^{(i-1)}\,{{\rm{e}}}^{{\rm{ika}}},\,(i=2,\,\ldots \ldots ,\,{\rm{n}}+1)$$

where, V_2R_ are the state vectors at each two consecutive unit cells (*i*) and (*i* − 1), respectively.

Generally, the wavenumber k is a complex number and it can be written in the following form

7 $${\rm{k}}={{\rm{k}}}_{{\rm{real}}}-{{\rm{ik}}}_{{\rm{imaginary}}}.$$

where k_real_ and ${{\rm{k}}}_{{\rm{imaginary}}}$ are the real and positive part of wavenumber, respectively.

Based on this relation, the complex conjugate pairs of the wavenumber will cause in an attenuating of the wave solutions within the band gaps. Therefore, we have two cases of frequency ranges as follow,

Firstly, if k = k_real_ and k_real_ > 0

By using Eq. (7),

8 $${{\rm{V}}}_{2{\rm{R}}}^{({\rm{i}})}={\,V}_{2{\rm{R}}}^{({\rm{i}}-1)}\,{e}^{{\rm{i}}|{{\rm{k}}}_{{\rm{real}}}|{\rm{a}}}.$$

From Eq. (8), the displacement and stress at unit cells (*i*)th and (*i* − 1)th differ only by a phase factor ${{\rm{e}}}^{{\rm{i}}|{{\rm{k}}}_{{\rm{real}}}|{\rm{a}}}$. This case indicates that the waves are permitted to propagate through the structure at frequency ranges belonging to these classes of frequencies which are called pass-band.

Secondly, if ${\rm{k}}={\mathrm{ik}}_{{\rm{imaginary}}}$ and ${{\rm{k}}}_{{\rm{imaginary}}} < \,0$

By using Eq. (7),

9 $${{\rm{V}}}_{2{\rm{R}}}^{({\rm{i}})}={\,V}_{2{\rm{R}}}^{({\rm{i}}-1)}\,{e}^{-|{{\rm{k}}}_{{\rm{imaginary}}}|{\rm{a}}}.$$

Equation (9) indicates that the displacement and stress at unit cells (*i*)th and (*i* − 1)th don’t have a phase difference. In addition, there is a spatial exponential attenuation in a magnitude of strength proportional to $|{{\rm{k}}}_{{\rm{imaginary}}}|$. Therefore, the wave motion cannot occur through the structure at frequencies ranges within these classes of frequencies which are called stop-bands or band gaps^51^.

All propagating modes of the wave will be the same in the first irreducible Brillouin zone (IBZ) due to the periodicity of the structure^52^. This means that these solutions are physically identical and allows to set the range of the independent values of q within the first Brillouin zone. The limits of the Brillouin zone is corresponding to $\mbox{--}{\rm{\pi }}\le {\rm{\zeta }}\le {\rm{\pi }}$. In Fig. 4(b), there are some frequencies ranges are associated with pass-band regions (weight color). These ranges corresponding to real-valued wave number and in which the elastic waves propagate freely through the structure.

The phononic band gap width in the dispersion curve in Fig. 4(b) is equal to the value 1.5132 × 10^5^ Hz and the center of the band gap is located at the value 75663 Hz. Also, the phononic band gap width (the lowest transmission intensity in the frequency spectrum) in the transmission curve in Fig. 4(a) is equal to the value 1.4642 × 10^5^ Hz and the center of the band gap is located at the value 73210 Hz. The small difference between these values is due to that the transmission spectrum must be plotted between two-semi-infinite materials, which affect the formation of phononic band gaps. Hence, the two Fig. 4(a,b) are almost compatible with the phononic band gap frequency range.

### Effect of porosity ratio on the phononic band gap of PSiC PnC

To study the porosity ratio effect on the phononic band gaps, assume a one unit cell and the thickness of each material was maintained at the value 3 mm. The first material is considered to be the PSiC_1_ with porosity ratio 14.41% that used in the previous calculations. The second one PSiC_x_ is considered at different porosities. The subscript  ×  refers to the porosity ratio values 17.60, 20.87, 23.77, and 31.65%, respectively. Table 2 shows the acoustic parameters of the materials PSiC_1_ and PSiC_x_.

**Table 2:** PSiC_1_ and PSiC_X_ acoustic parameters.

Material	Porosity ratio p (%)	Density (kg/m^3^)	Longitudinal sound speed (m/s)	Thickness (mm)
PSiC_1_	14.41	2550	9870	3
PSiC_x_	17.60	2450	9555	3
	20.87	2350	9360	3
	23.77	2260	9250	3
	31.65	2030	7990	3

**Figure 5:**
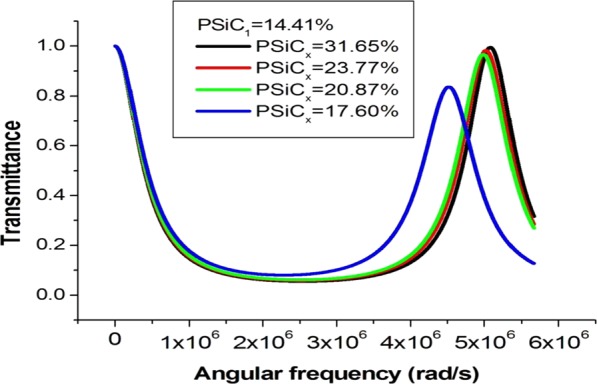
The transmission of a (PSiC_1_/PSiC_x_)^1^ PnC with porosity ratios PSiC_1_ = 14.41, 17.60, 20.87, 23.77 and 31.65%.

As the porosity ratio of PSiC_x_ material increases, the right edge of the phononic band gap red shift toward longer frequencies as shown in Fig. 5. With more increment in the porosity ratio of PSiC_x_, the entire frequency spectrum will be wider and behaves as a complete band gap due to the increment of the acoustic mismatch between the constituent materials. Also, increasing the porosity ratio of the second material PSiC_x_ means the number of voids will increase as well, which makes the second material less hardness. This means the porosity ratio has a vital role on the band gap formation, width and frequency range. The porosity increment of a structure causes a widening in the band-gaps^53^.

On the other hand, the conventional materials such as Al and steel have constant acoustic parameters (Young’s modulus, density and sound speed). Hence, the unique combination between the mass density and high elastic constant cannot be obtained with the conventional materials. Therefore, it is very difficult to control the phononic band gap.

### Local resonant PSiC PnC structures

#### Binary PSiC PnC structure

**Figure 6:**
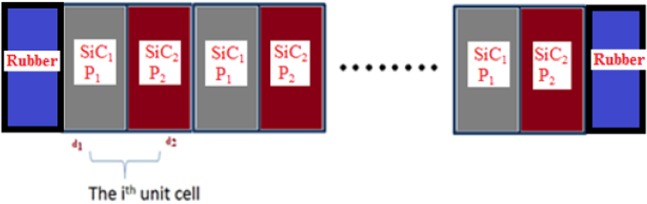
A schematic diagram of a 1D binary porous PnC consists of rubber /(PSiC_1_/PSiC_1_)^3^/rubber.

**Figure 7:**
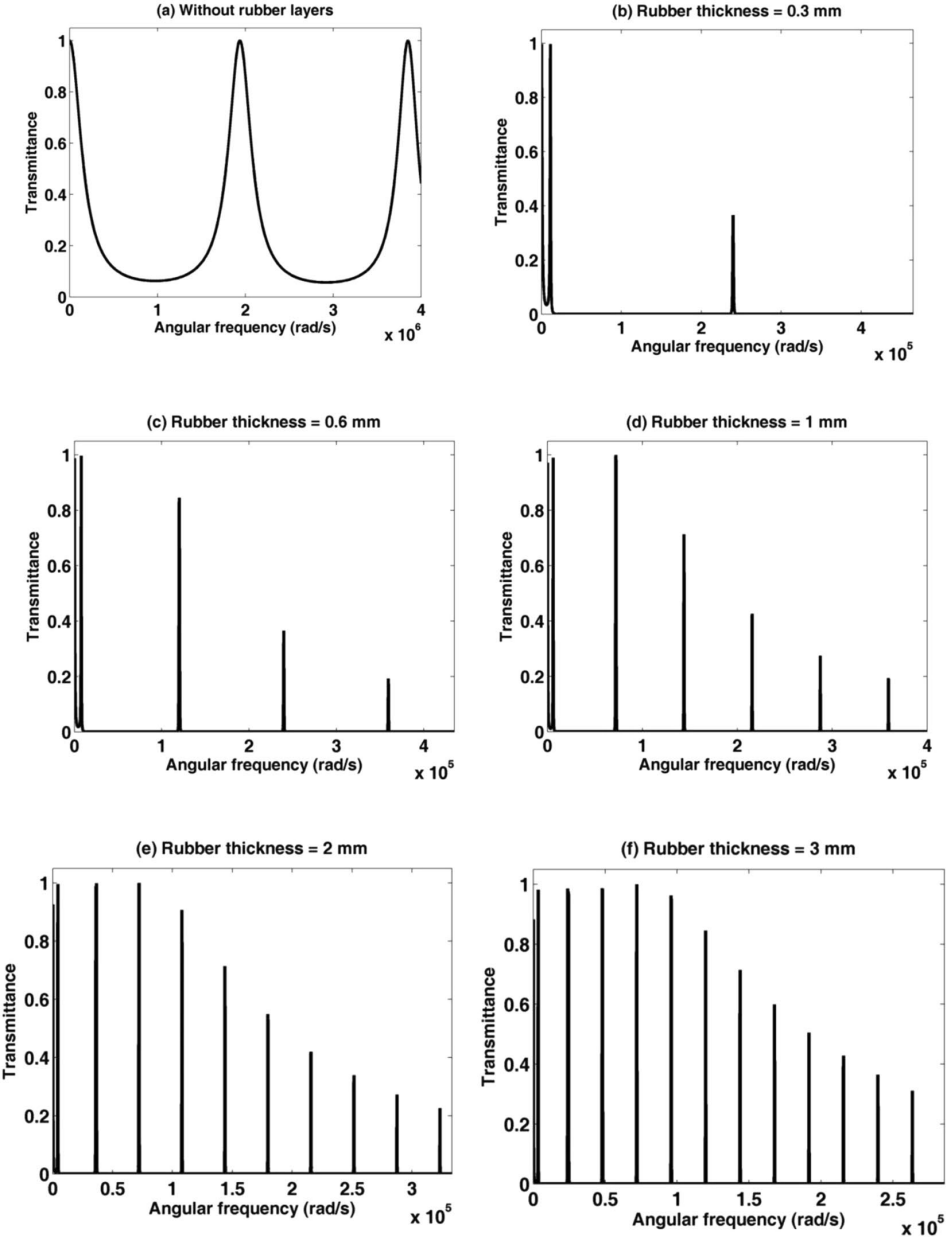
The transmission spectrum of a rubber/(PSiC_1_/PSiC_2_)^3^/rubber PnC with different rubber thicknesses (**a**) 0 mm, (**b**) 0. 3 mm, (**c**) 0.6 mm, (**d**) 1 mm, (**e**) 2 mm and (**d**) 3 mm.

Consider a 1D binary PSiC PnC sandwiched between two rubber layers; rubber/(PSiC_1_/PSiC_2_)^3^/rubber as shown in the schematic diagram in Fig. 6. The porosity ratios of PSiC_1_ and PSiC_2_ are 14.41% and 31.65%, respectively. The thickness of the rubber layers changes from 0 to 3 mm. As seen in Fig. 7, by bonding two rubber layers at the two opposite sides of the (PSiC_1_/PSiC_2_)^3^ PnC, many band gaps appeared for the incident acoustic waves. The numbers of these bands increase from 2 to 12 with increasing the thickness of each rubber layer from 0.3 to 3 mm. At the same time, transmission modes appeared and became very narrow and sharp. Also, the intensity of these transmission modes decreases from 1 to 0.2 with increasing the frequency values. The low elastic constants of the coating rubber layer besides the high elastic constant of SiC layers introduce these strong resonant modes and wide band gaps in the transmission spectrum of the PSiC PnC. These locally resonant structures do not behave as the previous results and cannot be explained by the Bragg’s diffraction theory^54^. As well-known, the phononic band gaps are formed due to the constructive interference depending on Bragg diffraction law. While the wave localization modes are explained by the local resonance mechanism and can be created inside the PnCs by different techniques like attaching a host soft material (rubber) to the PnC structure^19–21^. This is can be attributed due to the increment of the effective acoustic thickness of the structure after adding the two rubber layers. So the wave propagation characteristics can be tuned by adding and adjusting the thickness of the hosting rubber layers.

Many reported articles have studied the effect of the semi-infinite material on the local resonant modes and phononic band gap inside these structures despite this material is not a constituent material of unit cells. Arafa *et al*. observed that the bonded materials have an effect on the band structure in the 1D PnCs^55^. Also, Denghui and Zhiyu studied the locally resonant PnC double panel structure consisting of a 2D periodic array of cylindrical locally resonant pillar connected between upper and lower plates from rubber layer^56^. Similarly, Qian and Shi investigated the propagation of elastic waves in the locally resonant PnC connected between two plates from rubber^57^. Also, Oudich *et al*. designed a sonic band gap based on the locally resonant PnC with Pb capped^58^. Moreover, Zhengyou *et al*. used rubber as a host matrix in 3D systems to obtain the low frequency band gaps with a structure of small dimension^19^.

To get an intuitive explanation of the above results and the effect of semi-infinte material on the phononic band gaps, consider an acoustic wave propagates normally through a 1D-PnC, the band gaps frequency is given according to the Bragg law of the multilayered stack as follows^59,60^

10 $${{\rm{f}}}_{{\rm{B}}}=\frac{{\rm{m}}}{2}{(\frac{{{\rm{d}}}_{{\rm{a}}}}{{{\rm{V}}}_{{\rm{a}}}}+\frac{{{\rm{d}}}_{{\rm{b}}}}{{{\rm{V}}}_{{\rm{b}}}})}^{-1}.$$

Where, f_B_ is the frequency at the center of the band gap, m is the order number for the multiple band gaps, d_a_ and d_b_ are the thicknesses of the layers in each unit cell. V_a_ and V_b_ are the acoustic wave velocities in the layers. According to the above relation, it will be assumed the center of the first band gap should locate at the frequency value 9.4264 × 10^5^ Hz. However, we obtained low frequency band gaps based on the local resonance mechanism inside the PSiC PnC. In Fig. 7(e), the frequency at the center of the first band gap is located at the value 7957 Hz, which is 118 times smaller than the relevant above frequency. Hence, the obtained band gap is proportional to the audible frequency range. The PPnC is smaller in size many times than other low frequency 1D-PnC structures^21,54,61^. The conventional sonic crystals that could attenuate the audible frequencies are very large compared to the proposed PPnC. For example, the famous kinematic sculpture introduced by Martinez-sala *et al*. to the audible sound frequencies consists of an array of hollow steel cylinders with a diameter of 2.9 cm with a unit cell of 10 cm^62^. In addition to that, the reported PSiC 1D-PnCs at the micro-scale possess band gaps at the THz range^63^. Consequently, these micro-scale PSiC PnCs are very useful for acoustic isolation devices such as a gyroscope, narrow band-pass filter and resonator^64,65^. Moreover, the narrow transmission peaks created within the phononic band gaps opened an attractive area of PnCs in many important applications in the audible frequency range such as sensors, resonators, and wave multiplexers^66–70^.

#### Ternary PSiC PnC structure

**Figure 8:**
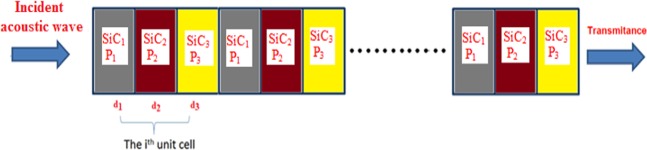
A Schematic diagram of a 1D ternary porous PnC consists of (PSiC_1_/PSiC_2_/PSiC_3_)^n^.

**Figure 9:**
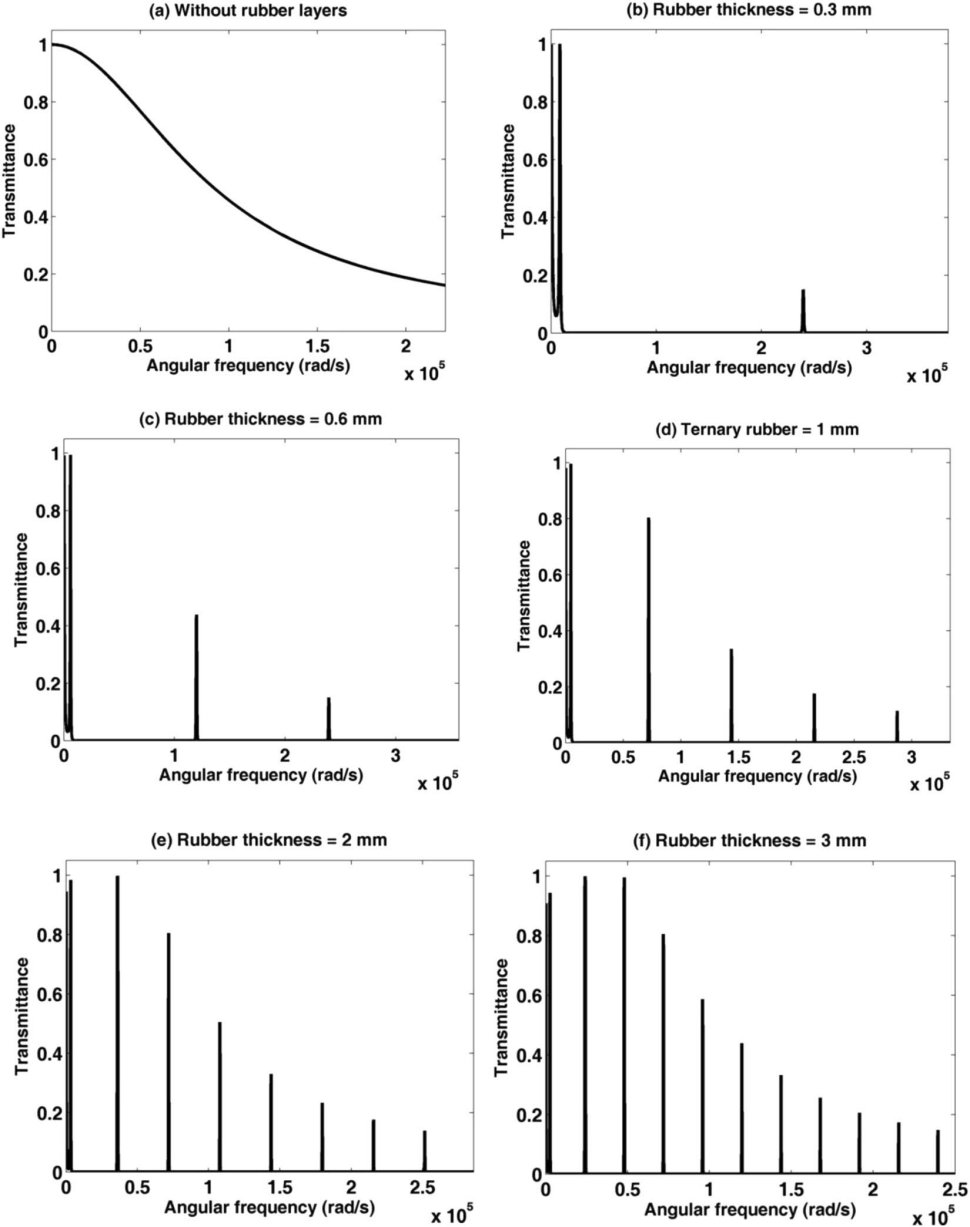
The transmission spectrum of a 1D ternary porous PnC: rubber/(PSiC_1_/PSiC_2_/PSiC_3_)^3^/rubber with different rubber thicknesses (**a**) 0 mm, (**b**) 0. 3 mm, (**c**) 0.6 mm, (**d**) 1 mm, (**e**) 2 mm and (**d**) 3 mm.

Consider a ternary PSiC PnC consists of three units and each unit cell is composed of three unit cells (i.e., nine layers). The third layer (PSiC_3_) immersed after the two layers that had been used in binary ones. The ternary PSiC PnC with the PSiC_3_ layer which has a porosity ratio of 20.875% and thickness 4 mm is shown in the schematic diagram in Fig. 8. A surprising behavior in the transmission spectrum can be seen in Fig. 9(a). Without adding rubber layers, the transmission of (PSiC_1_/PSiC_2_/PSiC_3_)^3^ PnC decreases with increasing the frequency value. Also, there are no band gaps present in the considered frequency regions. This strange behavior is because the three PSiC layers in the unit cell are close to each other in the elastic properties with a small acoustic mismatch between them, and thus the band gaps are disappeared. On the other side, there are many band gaps appeared for the acoustic waves when two rubber layers are bonded at the two sides of the ternary (PSiC_1_/PSiC_2_/PSiC_3_)^3^ PnC as seen in Fig. 9(b–f). Also, the number of these band gaps is larger than those obtained by the binary PSiC PnC. Moreover, the number of transmission modes increases with increasing the thickness of each rubber layer as in the case of binary PnC (Fig. 7). In addition to that, the intensity of these transmission modes decreases with increasing the frequency value.

To compare more between binary and ternary PSiC PnCs, for the case of rubber thickness = 2 mm, the number of transmission modes for the binary system (9 modes) is higher than that for the ternary system (7 modes). The small number of resonant modes in the ternary structure is due to that three materials have two interfaces in the ternary structure rather than one interface in the binary crystal. Therefore, the ternary structures are more dispersive for waves than the binary one as a result of increasing the heterogeneity in the ternary structure Also, the width of the band gap for ternary is higher than for the binary at the same thickness of rubber layers.

## Conclusions

We have demonstrated theoretically by using the TMM the formation of phononic band gaps in the audible frequencies depending on the porous SiC multilayer structure. A large number of acoustic resonant modes appeared when the structure sandwiched between rubber layers. An interesting interaction occurred between the extraordinary mechanical properties of porous SiC and the local resonance phenomenon caused by the rubber layers. Such interaction resulted in breaking the conventional Bragg law $1/{\rm{\omega }}\propto {\rm{\lambda }}\propto {\rm{a}}$, which leads to the formation of low frequency band gaps in small size structures compare with many previous studies.

A total wave reflection occurred in these 1D layered composites besides localized resonant modes with certain tunable sonic frequencies appeared. Acoustic resonant modes and band gap width can be tuned at different frequencies by changing the thickness, number and porosity of the rubber layers. Furthermore, we have performed a comparison between the effects of binary and ternary structures on the transmission spectrum of the PSiC 1D-PnC. From the above results, the proposed porous SiC PnC structures will promote many engineering applications in the low frequency range. Such resonant PSiC PnC behaves as a Metamaterial with effective negative elastic constants. It would be interesting to realize the proposed structures and to test them for noise harnessing devices.
